# Does severe mucositis impair oncological outcome in head and neck cancer patients? A pooled analysis of two prospective studies with long-term follow-up

**DOI:** 10.1186/s12885-025-14293-8

**Published:** 2025-05-21

**Authors:** Tanja Sprave, Jörg Sahlmann, Andreas R. Thomsen, Diana Klein, Henning Schäfer, Raluca Stoian, Vivek Verma, Anca-Ligia Grosu, Elsa Beatriz Monroy Ordonez

**Affiliations:** 1 Department of Radiation Oncology, University Hospital of Freiburg, Robert-Koch-Strasse 3, Freiburg, 79106 Germany; 2 German Cancer Consortium (DKTK) Partner Site Freiburg, German Cancer Research Center, Freiburg, Germany; 3 Faculty of Medicine, University of Freiburg, Freiburg, Germany; 4 Institute for Medical Biometry and Statistics, Medical Center - University of Freiburg, Faculty of Medicine, University of Freiburg, Freiburg, Germany; 5 Institute for Cell Biology (Cancer Research), University Hospital Essen, University of Duisburg-Essen, Essen, Germany; 6 Department of Radiation Oncology, The University of Texas MD Anderson Cancer Center, Houston, TX USA

**Keywords:** Head and neck cancer, Radiation therapy, Mucositis

## Abstract

**Background:**

Oral mucositis (OM) is a frequently reported radiotherapy (RT)-induced acute toxicity in head and neck (H&N) cancer patients. Severe OM may be a dose-limiting condition, which can affect oncological outcomes. Therefore, we conducted a pooled analysis of two prospective studies with long-term follow-up to evaluate the impact of grade ≥3 OM on outcomes.

**Methods:**

This pooled analysis included 253 H&N cancer patients who received primary definitive or adjuvant chemoradiotherapy at the University of Freiburg Medical Center**.** Kaplan–Meier analyses with log-rank tests stratified for the presence of grade 3 OM were performed for overall survival (OS), local recurrence-free survival (LRFS), and distant-metastasis free survival (DMFS). Univariate Cox proportional hazards regression was performed to identify prognostic factors for OS, LRFS, and DMFS.

**Results:**

The majority of participants had locally advanced disease: UICC stage IVA in 157 (62.1%) and IVB in 31 (12.3%). During treatment, 168 (66.4%) participants developed grade 3 OM. After a median follow-up of 73.6 months, the median OS was 64.6 months (95% CI, 47.6–83.7), and the median LRFS and DMFS had not yet been reached.

Advanced disease stages had a significant impact on OS as follows: UICC IVb vs. I, HR 4.62 (95%-CI: 1.364–5.637, SE 0.6, *p* = 0.014) and UICC IVc vs. I, HR 9.01 (95%-CI: 1.500–54.1643, SE = 0.9, *p* = 0.016). Previous surgery also has a significant impact on OS with an HR 0.65 (95% CI: 0.440–0.948, SE 0.2, *p* = 0.026). RT duration also showed a significant impact on OS with HR 1.03 (95% CI: 1.002–1.067, SE = 0, *p* = 0.040). For LRFS, prior surgery had a significant impact with an HR of 0.46 (95% CI: 0.247–0.857, SE 0.3, p = 0.014). Furthermore, the cumulative RT dose had a measurable impact on LRFS with HR 1.10 (95% CI: 1.022–1.189, SE = 0.03, *p* = 0.012). Smoker status showed a significant effect on DMFS with an HR 3.29 (95% CI: 1.090–9.872, SE 0.561, *p* = 0.034). The presence of grade 3 OM has no significant impact on LRFS, OS, or DMFS.

**Conclusions:**

Severe acute grade 3 OM shows no long-term impact on oncological endpoints. Validation in larger multicenter cohorts is recommended.

**Supplementary Information:**

The online version contains supplementary material available at 10.1186/s12885-025-14293-8.

## Introduction

For early-stage head and neck cancer (H&N), transoral surgery is usually the recommended primary treatment for lesions in the oral cavity, oropharynx, and larynx. Alternatively, definitive radiation therapy (RT) with or without concomitant chemotherapy may be used [1]. Primary surgical treatment is recommended for T3/T4 carcinomas of the oral cavity, T4 carcinomas of the larynx, and some hypopharyngeal carcinomas. However, advanced oropharyngeal lesions (regardless of HPV status) are currently treated with definitive chemoradiotherapy (CRT) [1]. For nasopharyngeal carcinoma, personalized approaches such as more intensive chemotherapy, induction, and adjuvant therapy are recommended [2]. Oral mucositis (OM) is an expected complication of cancer treatment in H&N patients, and the most frequently reported RT-induced acute toxicity [3, 4]. OM (an inflammatory condition) is characterized by erythema, tissue breakdown, and/or pain (grades 1–2), and can escalate to severe ulceration (grade 3 or higher). The overall incidence rate for all grades of OM ranges from 60–100% [5, 6]. Additionally, the accompanying deterioration in quality of life frequently leads to treatment interruptions [7, 8]. The resulting prolongation of overall treatment time can compromise treatment outcomes [9–12]. In particular, severe grade ≥3 OM is associated with a high symptom burden, hospitalization, and high resource utilization [4].

The impact of clinical and radiotherapy-specific parameters on OM has been well studied [13, 14]. Despite the widespread use of image-guided intensity modulated RT (IMRT) technique, the incidence of severe mucositis remains high, in part because treatment volumes still include large portions of the uninvolved mucosa, especially for locally advanced disease [3, 4, 6, 15, 16]. The intrinsic and extrinsic sensitivity to RT of the mucosa is the subject of active investigation [17–22].

However, isolating the effect of severe OM on oncological endpoints has not been sufficiently elucidated, especially using prospective data. Therefore, we conducted a pooled analysis from two prospective studies to examine whether the occurrence of grade 3 OM has an impact on survival and other oncologic outcomes.

## Materials and methods

This pooled analysis included H&N cancer patients who received primary definitive or adjuvant chemoradiotherapy between 2008 and 2022 within two prospective trials conducted at the University of Freiburg Medical Center.

One of these prospective studies (ZISStrans-study) included H&N cancer patients treated between 2017 and 2022. The aim of this study was to analyze the role of oral keratinocytes in predicting severe OM. Specifically, the growth of oral keratinocytes in vitro was demonstrated and the prediction of the oral mucosa was prospectively assessed [6, 22, 23]. 102 patients were recruited for the collection of healthy gingival tissue before the start of the (C)RT, of whom 18 were screening failures and study dropouts. A further 21 participants did not receive a biopsy for various reasons. Therefore, an oral microbiopsy of the healthy mucosa was obtained in 63 participants. A detailed report of the biopsy procedure has been published elsewhere [23]. Thus, 102 patients from ZISStrans-study were included in this pooled analysis.

The other study included H&N cancer patients treated between 2008 and 2015. The primary objective of this study was to analyze the value of protein profiles in blood serum and saliva for predicting severe OM. For our pooled analysis, 151 patients were included from this study. Due to incomplete records regarding the administration of chemotherapy in this study, the chemotherapy was not specified for pooled data. All procedures were approved by the Ethics Committee of the University of Freiburg (vote ETK-FR 449/16, amended by vote ETK-FR 413/17, and ETK-FR 30/10). Written informed consent was obtained in all patients. All personal data and biopsy samples were pseudonymized. All patients were discussed in a multidisciplinary tumor board. All carcinomas were confirmed by biopsy. Based on imaging and pathology, tumor nomenclature was established according to the 8 th edition of the TNM classification of malignant tumors of the UICC. Before a decision on multimodality treatment was made, staging by MRI and/or CT of the thorax was performed to exclude distant metastases. Prior to therapy, all patients received a dental examination and, if necessary, focal treatment or extraction. Individuals with a smoking history of at least 10 pack-years were considered as smokers.

Systemic therapy was applied according to current guidelines and recommendations of the tumor board; in brief, definitive (chemo)radiotherapy (CRT) was recommended for locally advanced and non-resectable tumors and was also influenced by age. Adjuvant cases were eligible for CRT based on surgical pathological findings.

The standard dose for definitive CRT was 70 Gy EQD2 to the primary tumor region, whereas patients undergoing adjuvant RT received 60–66 Gy EQD2 to the tumor cavity. The vast majority of RT was performed using intensity-modulated radiotherapy (IMRT) or helical IMRT such as tomotherapy. CT-based (Brilliance, CT Big Bore, Philips, Cleveland, OH, USA) three-dimensional treatment planning was performed (Oncentra MasterPlan, Nucletron, Veenendaal, The Netherlands; and Eclipse™ planning systems, Varian Medical Systems, Palo Alto, CA, USA), using individually collimated portals (6 or 18 MV; Synergy; Elekta, Crawley, United Kingdom), IMRT, or volumetric modulated arc therapy (VMAT) were used. Image-guided RT was performed for all patients; since 2019, RT was performed using Surface Guided RT (C-RAD, Catalyst, C-RAD AB, Uppsala, Sweden). Due to unplanned patient- and treatment-related interruptions during RT, additional compensatory fractions were applied to improve individual tumor response, resulting in an increase in the cumulative dose outside the standard dose. The additional compensatory dose was calculated using a fractionation factor $\frac{a}{\beta }=10$ Gy for fast-growing H&N tumors. The biological dose of 0.9 Gy^−1^ [24] (range 0.5–1.2) was assumed for the compensation of ongoing replication of the tumor cell [25–27].

The main focus of both trials was a systematic recording of mucositis. For this purpose, a clinical examination of the patients was performed twice a week by a specially trained examiner. This involved systematic examination of the nine regions of the oral mucosa: hard and soft palate, left and right tongue, floor of the mouth, upper and lower lip, as well as left and right buccal regions. World Health Organization scores for OM were used. In addition, local discomfort and its impact on daily activities were assessed by a questionnaire. During the (C)RT, all patients performed standardized daily oral care consisting of mouth rinsing 3–4 times per day, use of a soft toothbrush, and regular fluoridation of the teeth.

All patients were monitored by a surgeon and radiation oncologist every three to six months for the first two years, followed by annual visits thereafter. Acute side effects (until 90 days post-therapy) were evaluated according to the Common Terminology Criteria for Adverse Events version 5.0 (CTCAE v.5). Late toxicity was judged using the modified Late Effects in Normal Tissues criteria (subjective, objective, management, and analytic, LENT-SOMA). All local recurrences were confirmed histologically.

### Statistical analysis

Descriptive statistics are reported as a mean, median (range), and frequencies. The overall survival (OS) was estimated from the start of RT until death from any cause. Whereas local recurrence free survival (LRFS) and distant metastasis free survival (DMFS) were estimated from the start of RT until local or distant oncologic progression, as defined by Response Evaluation Criteria in Solid Tumors 1.1, respectively. For OS, patients were censored at the last known date of documented survival, and for LRFS and DMFS, at the last oncology follow-up without local or distant progression, respectively. Kaplan–Meier analyses with log-rank tests stratified for the presence of grade 3 OM were performed for OS, LRFS, and DMFS.

Univariate and multivariate Cox proportional hazards regression were performed to identify prognostic factors for OS, LRFS, and DMFS, including demographic and clinical variables relevant to survival. The following variables were included: gender, age (< 65 vs. ≥ 65 years), smoking status, tumor localization, UICC stage, tumor grading, prior surgery, RT duration, RT interruptions, RT duration ≥ median vs. < median, and RT total dose. Hazard ratios (HR) with 95% confidence intervals (95% CI) for survival were reported, calculated in months.

All tests and CIs were 2-sided, with statistical significance set at α < 0.05. Statistics were performed with SPSS version 29 (IBM, Armonk, NY, USA) and the open-source statistical software environment R (version 4.4.0).

## Results

A total of 253 H&N cancer patients from two prospective studies treated with primary or adjuvant radiotherapy RT were included. Patient and treatment characteristics are shown in Table 1. Cancers were situated in the oropharynx (*n* = 118, 46.6%), hypopharynx (*n* = 64, 25.3%), and the oral cavity (*n* = 37, 14.6%). The majority of participants had locally advanced disease: IVA in 157 (62.1%) and IVB UICC stage in 31 (12.3%). The majority of patients 202/253 (79.8%) were treated using IMRT. During treatment, 168 (66.4%) participants developed grade 3 OM. No patient experienced a grade ≥ 4 event. In the group with grade 3 OM, 116 (69%) patients were < 65 years and 52 (31%) ≥ 65 years; 41 (24%) were female and 127 (76%) male. Grade 3 OM developed in 112 (67%) patients after definitive RT and 56 (33%) following adjuvant RT. The median RT duration was 49 days (min 34, max 71) in the group with grade 3 OM and 48 days (min 11, max 74) in the group with grade 1–2 OM. Correspondingly, the mean RT dose in the group with grade 3 OM was 67.3 Gy (standard deviation (SD) ± 4.8, min 50, max 81) vs. 65.2 Gy (SD ± 8.8, min 20, max 72) group without severe OM. For the entire cohort, the median RT duration was 48 days (95% CI: 48; 49) (Fig. 3).

**Table 1:** Main characteristics of patients, tumor and therapy details of head and neck cancer patients treated by (chemo)radiotherapy ((C)RT) in our institution between 2006 and 2022 (*n* = 253). Staging of primary head and neck cancer was based on the 8 th Edition of the UICC TNM classification

Total *N* = 253			
Oral mucositis grade 3		No (*N* = 85)	Yes (*N* = 168)
Age at primary diagnosis [years]	n valid/N (%)	85/85 (100%)	168/168 (100%)
Age at primary diagnosis [years]	Mean ± SD	60.07 ± 8.894	60.91 ± 8.306
	Min	38.1	42
	Median (Q1, Q3)	60.0 (53.0, 65.0)	60.2 (55.0, 67.0)
	Max	81	84.2
Age categorised	< 65 years	62 (73%)	116 (69%)
	≥ 65 years	23 (27%)	52 (31%)
Gender	Female	15 (18%)	41 (24%)
	Male	70 (82%)	127 (76%)
Smoker	No	10 (19%)	56 (42%)
	Yes	43 (81%)	77 (58%)
	Missings	32/85 (38%)	35/168 (21%)
Tumor localisation	CUP	3 (4%)	1 (1%)
	Hypopharynx	31 (36%)	33 (20%)
	Larynx	6 (7%)	22 (13%)
	Oral cavity	9 (11%)	28 (17%)
	Oropharynx	36 (42%)	82 (49%)
	Nasopharynx	0 (0%)	2 (1%)
UICC stage	I	3 (4%)	11 (7%)
	II	8 (9%)	15 (9%)
	III	9 (11%)	17 (10%)
	IVA	51 (60%)	106 (63%)
	IVB	14 (16%)	17 (10%)
	IVC	0 (0%)	2 (1%)
Grading	1	1 (1%)	7 (4%)
	2	50 (62%)	95 (59%)
	3	30 (37%)	58 (36%)
	4	0 (0%)	2 (1%)
Surgery	No	54 (64%)	112 (67%)
	Yes	31 (36%)	56 (33%)
RT duration [days]	n valid/N (%)	85/85 (100%)	168/168 (100%)
	Mean ± SD	46.8 ± 7.73	48.2 ± 5.78
	Min	11	34
	Median (Q1, Q3)	48 (43, 50)	49 (44, 51)
	Max	74	71
RT total dose [Gy]	n valid/N (%)	85/85 (100%)	85/85 (100%)
	Mean ± SD	65.222 ± 8.730	67.283 ± 4.779
	Min	20	50
	Max	72	81

*Abbreviations*: *SD* standard deviation, *UICC TNM* tumor staging system according to Union for International Cancer Control

**Fig. 1:**
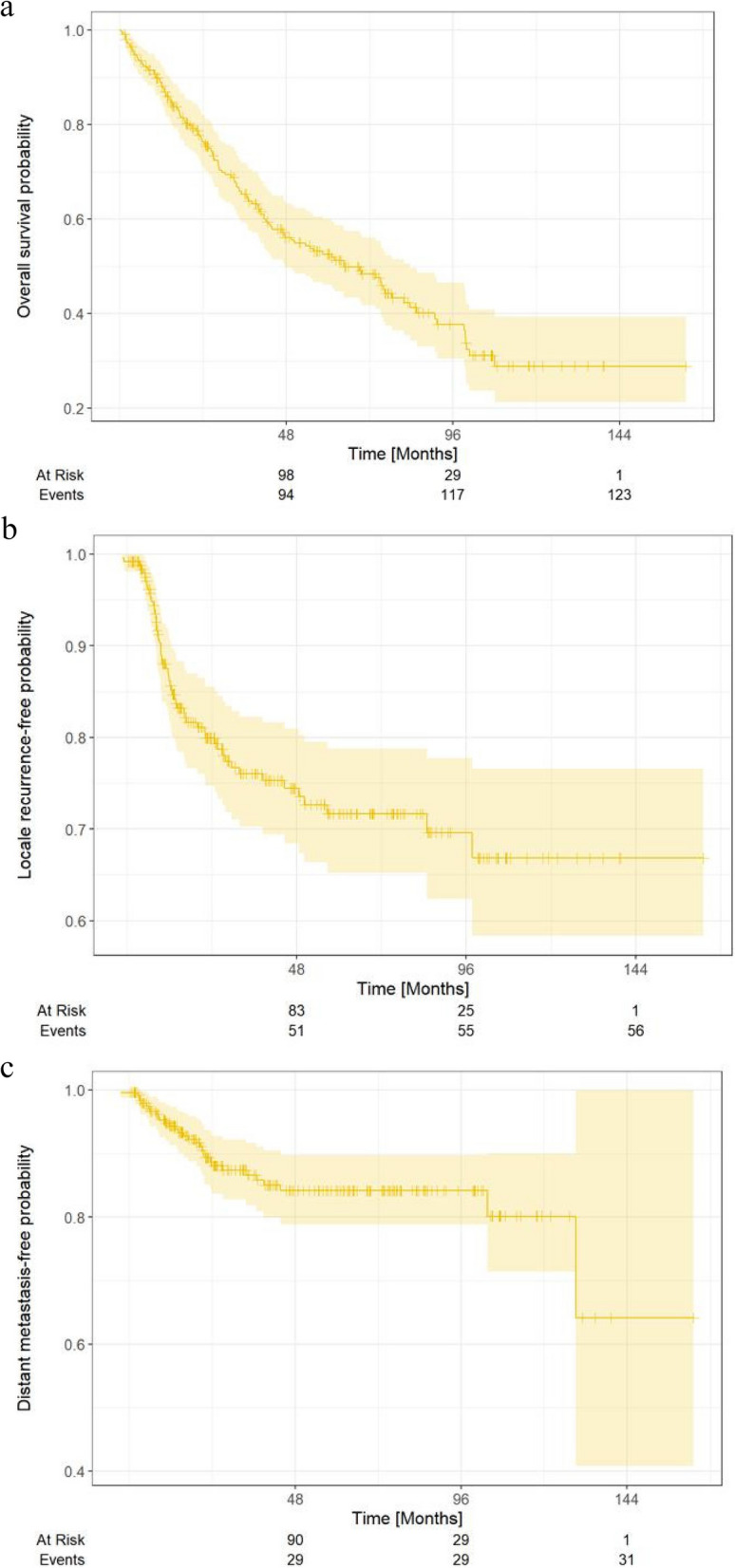
Kaplan-Meier curves regarding overall survival (OS), local-recurrence free survival (LRFS), and distant metastasis free survival (DMFS)

**Fig. 2:**
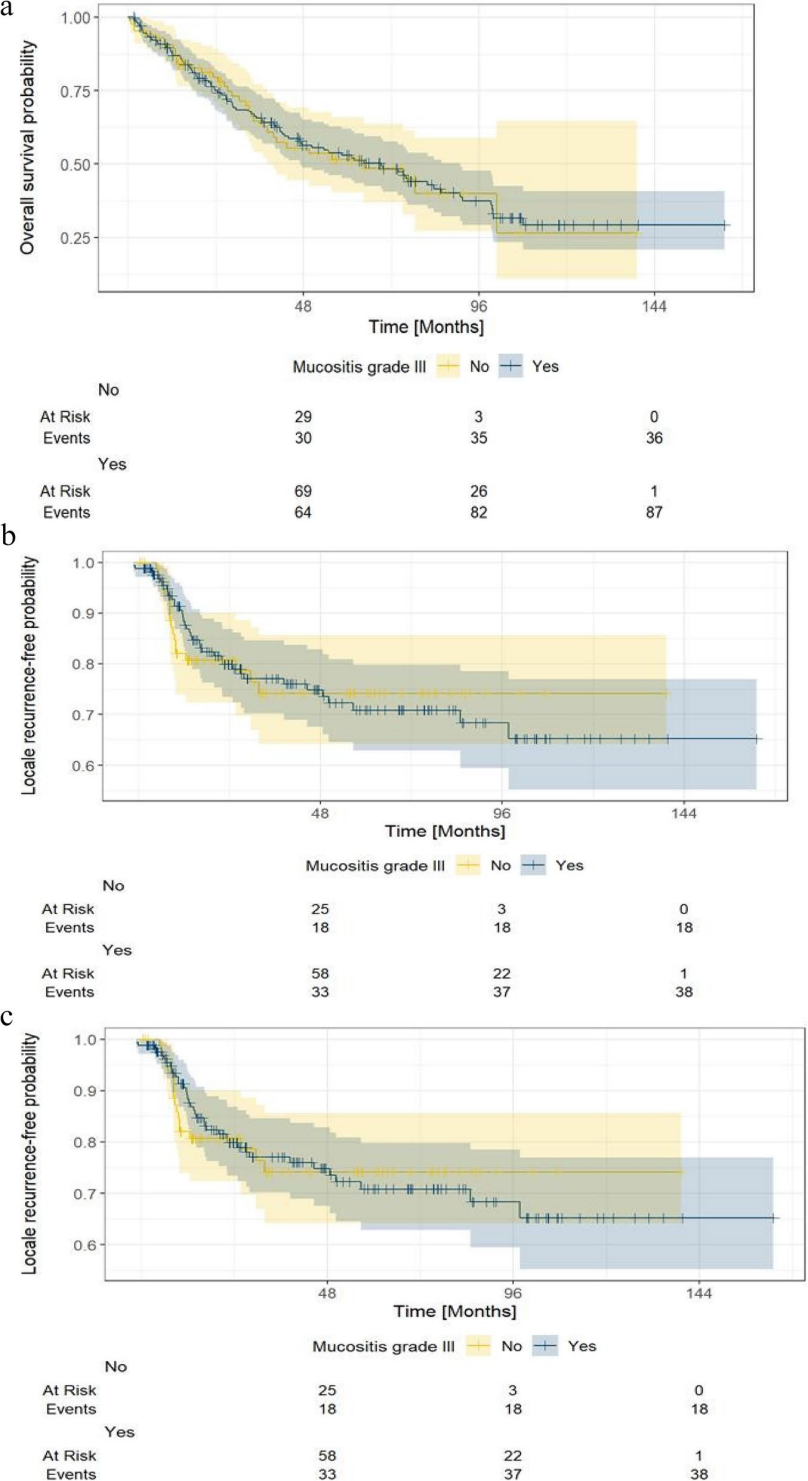
Kaplan-Meier curve stratified by mucositis grade 3 regarding overall survival (OS), local-recurrence free survival (LRFS), and distant metastasis free survival (DMFS)

**Fig. 3:**
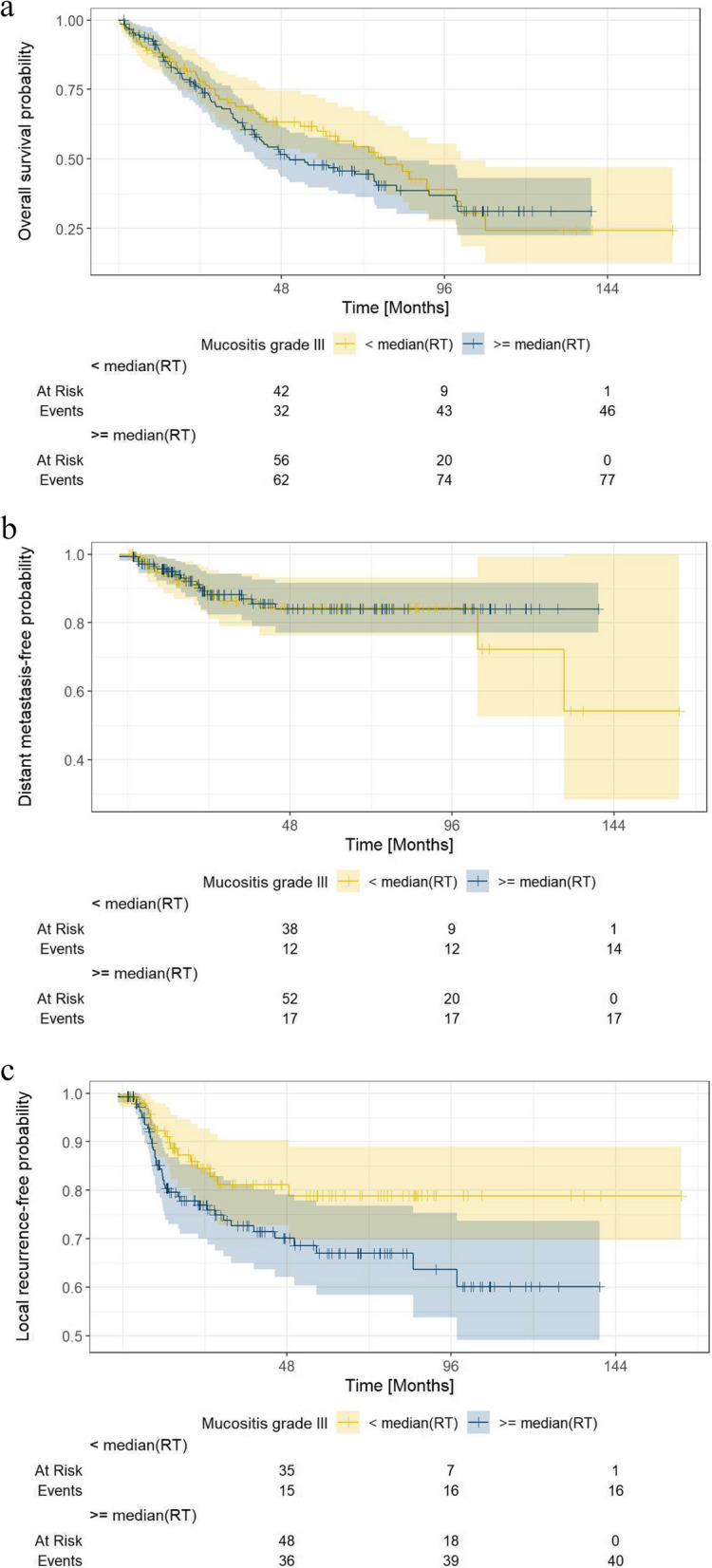
Kaplan-Meier curve stratified by mucositis grade 3 and radiotherapy (RT) duration < median vs. ≥ median regarding overall survival (OS), local-recurrence free survival (LRFS), and distant metastasis free survival (DMFS)

After a median follow-up of 73.6 months (95% CI, 64.9–78.7), the median OS was 64.6 (95% CI, 47.6–83.7) months (Fig. 1a), whereas the median LRFS and DMFS had not yet been reached (Fig. 1b, c). Kaplan–Meier analyses for OS, LRFS, and DMFS stratified by the presence of grade 3 OM as well as RT duration ≥ median vs. < median, are provided in Figs. 2 and 3.

Univariate Cox-regression analysis evaluated the impact of demographic and tumor characteristics (shown in Table 1) on OS, LRFS, and DMFS. Advanced disease stages had a significant impact on OS as follows: UICC IVb vs. I, HR 4.62 (95%-CI: 1.364—15.637, SE 0.6, *p* = 0.014) and UICC IVc vs. I, HR 9.01 (95%-CI: 1.500—54.1643, SE = 0.9, *p* = 0.016) (Table 2). Previous surgery also had a significant impact on OS with an HR 0.65 (95% CI: 0.440—0.948, SE 0.2, *p* = 0.026). RT duration and RT interruption (yes vs. no) also showed a significant impact on OS with HR 1.03 (95% CI: 1.002—1.067, SE = 0, *p* = 0.040) and HR 2.02 (95% CI: 1.379—2.968) SE = 0.200 *p* < 0.001) respectively (Table 2). For LRFS, prior surgery (vs. no surgery) again had a significant impact with an HR of 0.46 (95% CI: 0.247—0.857, SE 0.3, *p* = 0.014). Furthermore, the cumulative RT dose had a measurable impact on LRFS with HR 1.10 (95% CI: 1.022—1.189, SE = 0.03, *p* = 0.012) (Table 2). Smoker status (vs. non-smoker) showed a significant effect on DMFS with an HR 3.29 (95% CI: 1.09—9.872, SE 0.561, *p* = 0.034). In the multivariate analysis, only the following independent variables had a significant impact on OS: UICC IVb vs. I with HR 3.78 (95% CI: 1.086–13.159, SE = 0.636, *p* = 0.037, prior surgery (yes vs. no) HR 0.624 (95% CI:0.392–0.993, SE = 0.237 *p* = 0.047), and RT interruption (yes vs. no) HR 2.572 (95% CI:1.526–4–334 SE = 0.266, *p* < 0.001) (Table 2).

**Table 2:** Univariate and multivariate Cox proportional hazards regression to identify prognostic factors for overall survival (OS), local recurrence-free survival (LRFS), and distant metastasis free survival (DMFS) including demographic and clinical variables

Univariate Cox-Regression: Overall Survival (OS)				
Variable	Hazard Ratio	95%-CI	SE	*p*-value
Age	1,00	0.980—1.021	0.011	0.981
Age ≥ 65 vs. < 65 years	1,17	0.796—1.713	0.196	0.428
Male vs. female	1,21	0.770—1.908	0.232	0.407
Smoker vs. No-Smoker	1,38	0.885—2.158	0.227	0.155
Oral cavity vs. oropharynx	0.74	0.367—1.501	0.359	0.407
Hypopharynx vs. oropharynx	1,22	0.800—1.850	0.214	0.36
Larynx vs. oropharynx	1,20	0.713—2.013	0.265	0.496
UICC II vs. I	1,15	0.288—4.600	0.707	0.843
UICC III vs. I	1,69	0.458—6.254	0.667	0.43
UICC IVa vs. I	2,34	0.738—7.396	0.588	0.149
UICC IVb vs. I	4,62	1.364—15.637	0.622	0.014*
UICC IVc vs. I	9,01	1.500—54.164	0.915	0.016 *
Tumor grading 2 vs. 1	3,51	0.859—14.302	0.717	0.08
Tumor grading 3 vs. 1	3,03	0.730—12.549	0.726	0.127
Tumor grading 4 vs. 1	1,94	0.176—21.386	1.225	0.589
Surgery yes vs. no	0.65	0.440—0.948	0.196	0.026 *
RT duration	1,03	1.002—1.067	0.016	0.040 *
RT total dose	0.98	0.951—1.019	0.018	0.374
RT interruption Yes vs. No	2.02	1.379—2.968	0.200	< 0.001*
RT duration > = median vs. < median	1.15	0.798—1.659	0.194	0.451
Univariate Cox-Regression: Local recurrence free survival (LRFS)				
Variable	Hazard Ratio	95%-CI	SE	*p*-value
Age	0.98	0.949—1.010	0.016	0.179
Age ≥ 65 vs. < 65 years	0.53	0.268—1.053	0.349	0.07
Male vs. female	1,54	0.752—3.133	0.364	0.239
Smoker vs. No-Smoker	1,91	0.947—3.854	0.358	0.071
Oral cavity vs. oropharynx	0.79	0.302—2.081	0.493	0.636
Hypopharynx vs. oropharynx	1,38	0.742—2.577	0.318	0.308
Larynx vs. oropharynx	1,34	0.600—2.980	0.409	0.477
UICC II vs. I	1,49	0.288—7.654	0.837	0.637
UICC III vs. I	0.84	0.140—5.013	0.913	0.846
UICC IVa vs. I	2,04	0.493—8.428	0.724	0.326
UICC IVb vs. I	1,58	0.306—8.144	0.837	0.586
UICC IVc vs. I	0	0—Inf	3,406.700	0.997
Tumor grading 2 vs. 1	0.84	0.256—2.745	0.605	0.772
Tumor grading 3 vs. 1	0.62	0.180—2.122	0.629	0.444
Tumor grading 4 vs. 1	0	0—Inf	3,650.086	0.996
Surgery yes vs. no	0,46	0.247—0.857	0.317	0.014 *
RT Duration	1,03	0.988—1.078	0.022	0.158
RT total dose	1,10	1.022—1.189	0.039	0.012 *
RT interruption Yes vs. No	0,81	0.406–1.598	0.349	0.563
RT duration > = median vs. < median	1,75	0.983–3.135	0.296	0.057
Univariate Cox-Regression: Distant metastasis free survival (DMFS)				
Variable	Hazard Ratio	95%-CI	SE	*p*-value
Age	1,004	0.963—1.046	0.021	0.862
Age ≥ 65 vs. < 65 years	0.88	0.391—1.959	0.412	0.746
Male vs. female	1,89	0.659—5.421	0.538	0.237
Smoker vs. No-Smoker	3,29	1.094—9.872	0.561	0.034 *
Oral cavity vs. oropharynx	2,43	0.879—6.719	0.519	0.087
Hypopharynx vs. oropharynx	2,18	0.930—5.131	0.436	0.073
Larynx vs. oropharynx	0,32	0.042—2.527	1,047	0.282
UICC II vs. I	0.348	0.058—2.086	0.914	0.248
UICC III vs. I	0.509	0.102—2.539	0.820	0.410
UICC IVa vs. I	0,45	0.131—1.540	0.628	0.203
UICC IVb vs. I	1,09	0.270—4.408	0.713	0.903
UICC IVc vs. I	0	0—Inf	4,702.860	0.997
Tumor grading 2 vs. 1	1,22	0.161—9.231	1.033	0.849
Tumor grading 3 vs. 1	1,18	0.151—9.304	1.052	0.872
Tumor grading 4 vs. 1	0	0—Inf	5,066.736	0.998
Surgery yes vs. no	0,96	0.459—1.987	0.374	0.902
RT Duration	0,99	0.929—1.056	0.033	0.770
RT total dose	1,03	0.954—1.118	0.041	0.453
RT interruption Yes vs. No	0,80	0.307–2.104	0.491	0.656
RT duration > = median vs. < median	0,87	0.428–1.786	0.364	0.712
Level'Nasopharynx,CUP', was omitted from tumor localisation for the purpose of this analysis				
Multivariate Cox-Regression: Overall Survival (OS)				
Variable	Hazard Ratio	95%-CI	SE	*p*-value
UICC II vs. I	0.96	0.271–3.900	0.714	0.956
UICC III vs. I	1,62	0.432–6.049	0.673	0.476
UICC IVa vs. I	2.2	0.679–7.118	0.599	0.189
UICC IVb vs. I	3.78	1.086–13.159	0.636	0.037*
UICC IVc vs. I	4.85	0.777–30.284	0.934	0.091
Surgery yes vs. no	0.624	0.392–0.993	0.237	0.047*
RT Duration	0.967	0.924–1.011	0.023	0.139
RT interruption Yes vs. No	2.572	1.526–4–334	0.266	< 0.001*
Multivariate Cox-Regression: Local recurrence free survival (LRFS)				
Variable	Hazard Ratio	95%-CI	SE	*p*-value
Surgery yes vs. no	0.69	0.285–1.665	0.45	0.408
RT total dose	1.07	0.960–1.184	0.053	0.22
Multivariate Cox-Regression:Distant metastasis free survival (DMFS)				
Variable	Hazard Ratio	95%-CI	SE	*p*-value
Smoker vs. No-Smoker	2.47	0.801–7.595	0.574	0.116
Oral cavity vs. oropharynx	2.57	0.776–8.533	0.612	0.122
Hypopharynx vs. oropharynx	1.98	0.678–5.769	0.546	0.212
Larynx vs. oropharynx	0.41	0.500–3.374	1.075	0.407
Level'Nasopharynx,CUP'was omitted from tumor localisation for the purpose of this analysis				

*Abbreviations*: *CUP* carcinoma of unknown primary, *UICC TNM* tumor staging system according to Union for International Cancer Control

The demographic and clinical parameters of the participants such as gender, age (< 65 vs. ≥ 65 years), UICC stage, tumor grading, smoking status, prior surgery, duration and total dose of RT had no significant impact on the occurrence of grade 3 OM (*p* > 0.05 for all) (Figure: S1).

## Discussion

The role of severe mucositis in long-term survival has not yet been thoroughly investigated, especially with prospective data. This could be because in clinical care, severe OM is perceived as a complex, multi-faceted toxicity – causing pain, odynophagia, as well as consistent malnutrition/dehydration, with the subsequent need for hospitalization. In this pooled analysis, we evaluated the long-term survival of patients with and without acute severe grade 3 OM. This pooled analysis was based on two prospective studies, which primarily addressed the specific recording of acute OM under RT [6, 22]. Our results suggested that therapy-induced grade 3 OM had no significant impact on OS, LRFS, and DMFS (Fig. 2a-c).

In our analysis, two-thirds of participants had locally advanced disease: IVA in 157 (62.1%) and IVB UICC stage in 31 (12.3%) (Table 1). The locally advanced stages were treated with extended radiation volumes and required a sufficient dose for optimal tumor control of up to 70 Gy, which in turn resulted in a high rate of acute grade 3 OM of 66.4% in our study. These results are similar to the findings of the cohort study by Iovoli et al. which found a rate of 62.5% severe OM in patients treated with IMRT. Another publication pertaining to probiotics on the occurrence, severity, and duration of severe OM showed an incidence of 54.2% in the placebo (non-probiotic) group [20]. In addition, there is insufficient information regarding possible associations between the timing of onset of mucositis and clinical endpoints [28, 29].

Up to 20% H&N cancer patients discontinue RT due to treatment induced toxicity [11, 20, 30–32] and receive an insufficient treatment [5, 33, 34]. In particular, RT interruption > 1 week increases the risk of locoregional recurrence up to 46% (6/13 patients), in UICC stage III-IV treated with definitive CRT after shot median follow-up of 12 months [11]. Despite the completion of definitive RT, the local recurrence rate is approximately 25% (151/608 patients) at 5 years [35]. In our study, after a median follow-up of 73.6 months (95% CI, 64.9–78.7), the median OS was 64.6 months (95% CI, 47.6–83.7) (Fig. 1a), and the median LRFS and DMFS had not yet been reached (Fig. 1b, c). Lohynska et al. reported a slightly worse median OS and disease-free survival (DFS) 45 and 41 months each, with median LRFS not having been reached in their publication of locally advanced H&N cancer status post definitive CRT [36]. The authors found that the RT treatment time extension from a mean of 48.6 to 50.2 days worsened survival in the groups with stage IV tumors [36]. In our study, the mean RT duration was similar 46.8 ± 7.73 SD days and 48.2 ± 5.78 SD days in the cohorts without severe OM and with grade 3 OM, respectively (Table 1). Our study supported the finding of Lohynska et al.: univariate Cox regression analysis showed that prolonging RT duration with mean of two days had a significant impact on OS with HR 1.03 (95% CI: 1.002–1.067, SE = 0.040, *p* = 0.040) (Table 2). Our findings of worse survival in advanced stages and after prior surgery is also concordant with other studies [9]. In general, surgical removal of operable and relatively smaller tumors (with presumably better oncological outcomes) is most often performed. Therefore, it is unclear whether the operation alone or a better prognosis of smaller tumors causes improved OS and LRFS. It is conceivable, that the patients who are more likely to experience serious events are probably the multimorbid patients who could die from other causes. Therefore, there are no differences in OS here, which is one more reason to continue to be aggressive with RT in these patients as well, rather than immediately follow a palliative approach. The lack of a difference in survival may be because the sole recording of toxicities does not say much about the outcomes, as they are not fatal or even only indirectly fatal. They can be indirectly fatal in terms of treatment breaks and such, but we have ways to compensate for almost all of those other factors.

Despite this pooled analysis from the large cohort, the limitations of these data should be noted. This analysis was conducted in only one institution and may not be representative or extrapolatable to the general population. The stratified Kaplan–Meier curves intersect (Fig. 2a-c), which may indicate a possible failure to meet the proportional hazard assumption. This also applies to the Cox regression analysis (Table 2). Thus, the interpretation of our results, notably the absence of an impact of grade 3 OM on survival, should be considered with caution and evaluated in larger collectives. Unfortunately, the recording of simultaneous chemotherapy was incomplete in our study, so systemic therapy was not considered in the evaluation and interpretation of our results. Further limitations of this analysis include the lack of information on therapy-related hospitalization, the total symptom burden and the exact location of the OM. Another limiting factor is the lack of information on HPV status, which is an important prognostic factor for OS. The strengths of our study consist of the prospective systematic recording of OM in a large collective and concordant results with the current literature.

It remains to be determined whether the onset of mucositis (early versus late) could have an influence on these parameters. We are currently investigating this aspect in our own retrospective and prospective patient cohorts.

## Supplementary Information

Additional file 1: Figure S1. The impact of the demographic and clinical parameters on the occurrence of grade 3 oral mucositis.

## Data Availability

The data used in this analysis are available with the authors’ permission.

## Abbreviations

CRT: Chemoradiotherapy
DMFS: Distant-metastasis free survival
Gy: Gray
H&N: Head and neck cancer patients
HPV: Human papillomavirus
IMRT: Intensity modulated radiotherapy
LRFS: Local recurrence free survival
OM: Oral mucositis
OS: Overall survival
RT: Radiotherapy
VMAT: Volumetric modulated arc therapy
