# Supplementary material for: Does severe mucositis impair oncological outcome in head and neck cancer patients? A pooled analysis of two prospective studies with long-term follow-up
Source: BMC Cancer. 2025 May 21;25:909. doi: 10.1186/s12885-025-14293-8 (PMC12096531; doi:10.1186/s12885-025-14293-8)
Supplement: Supplementary file 1 — Additional file 1: Figure S1. The impact of the demographic and clinical parameters on the occurrence of grade 3 oral mucositis. [file 12885_2025_14293_MOESM1_ESM.docx]

Covariates by mucositis grade III - Continuous variables

Age

RT duration

RT total dose

Covariates by mucositis grade III - Categorical variables

Age categorised

Gender

Smoking status

Tumor localisation

The level “Nasopharynx” was omitted for modelling purposes.

The level “Oropharynx” was consideres as the reference level


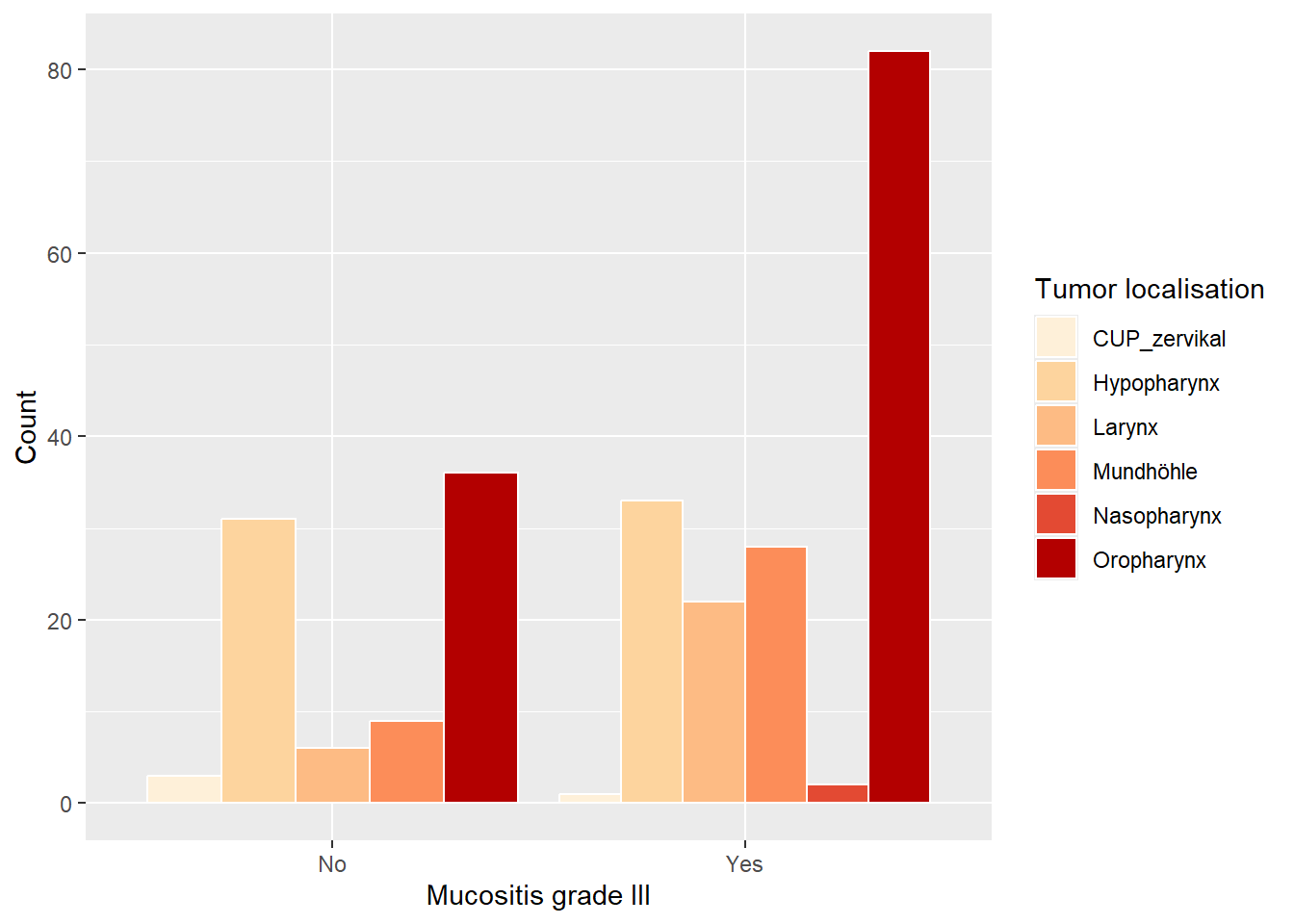


UICC stage

Grading

Surgery status
